# Monitoring the Prevalence and Distribution of Chytrid Fungus (*Batrachochytrium dendrobatidis*) in the Kihansi Spray Toad (*Nectophrynoides asperginis*) Population in the Kihansi Gorge Spray Wetlands, Tanzania

**DOI:** 10.1002/ece3.72873

**Published:** 2026-01-09

**Authors:** Devolent T. Mtui, Leonard J. Haule, Joseph O. Ogutu, Asa Preston, Josephine Braun, William D. Newmark, Edward M. Kohi, Juma Kimera, Mikidadi Mtalika, Hussein Adam, Samueli Mtoka, Felix Shayo, Julius D. Keyyu, Mariam R. Makange, Jean N. Hakizimana, Gerald Misinzo, Eblate E. Mjingo

**Affiliations:** ^1^ Tanzania Wildlife Research Institute Arusha Tanzania; ^2^ Biostatistics Unit, Institute of Crop Science University of Hohenheim Stuttgart Germany; ^3^ San Diego Zoo Wildlife Alliance, Amphibian Disease Laboratory Escondido California USA; ^4^ Natural History Museum of Utah, University of Utah Salt Lake City Utah USA; ^5^ Ministry of Natural Resources and Tourism Dodoma Tanzania; ^6^ SACIDS Foundation for One Health, Sokoine University of Agriculture Morogoro Tanzania; ^7^ Department of Veterinary Microbiology, Parasitology and Biotechnology, College of Veterinary Medicine and Biomedical Sciences Sokoine University of Agriculture Morogoro Tanzania

**Keywords:** *Batrachochytrium dendrobatidis*, captive breeding, chytrid fungus, Kihansi spray toad, spray wetlands, Udzungwa Mountains

## Abstract

Chytridiomycosis, caused by the fungus *Batrachochytrium dendrobatidis* (*Bd*), is fatal to some amphibian species, whereas others can carry the pathogen without developing disease. Among the vulnerable species is the Kihansi Spray Toad, *Nectophrynoides asperginis* , endemic to the Kihansi Gorge spray wetlands in Tanzania's Udzungwa Mountains. By 2009, chytridiomycosis had driven *N. asperginis* to extinction in the wild, though it survives in captivity. Re‐introduction efforts have faced difficulties, underlining the importance of understanding *Bd's* prevalence in the wild to guide re‐introduction programs. Twenty years after *Bd* was first detected in Tanzania's Kihansi Gorge spray wetlands, we re‐evaluated its prevalence and examined whether the pathogen was responsible for the 98% mortality observed among the 1000 captive‐bred *N. asperginis* released there in February 2022. In December 2022, time‐constrained surveys were conducted across three spray wetlands covering *N. asperginis* habitat. Amphibians were skin‐swabbed following a protocol developed by the University of California, resulting in two sets of 44 samples from seven species. These samples were analyzed for *Bd* presence using conventional and real‐time quantitative polymerase chain reaction, followed by nucleotide sequencing of PCR products. *Bd* was detected in 32% of samples, representing four out of the seven species: *Arthroleptides yakusini* (14%), *N. asperginis* (9%), *Ptychadena anchietae* (7%), and *Hyperolius substriatus* (2%). Whereas *N. asperginis* was severely affected, the other species did not show signs of the disease. The other three species, namely, *Hyperolius mitchelli, Afrixalus fornasinii*, *and Arthroleptis xenodactyloides*, were not detected with *Bd.* A lineage‐specific qPCR diagnostic test confirmed *Bd*‐CAPE as the cause of the mass deaths of the released toads. The continuing presence of *Bd*‐CAPE in the spray wetlands remains a barrier to the successful re‐introduction of *N. asperginis* , necessitating further experimentation to develop strategies for coexistence.

## Graphical abstract

How to manage the continued impact of Chytrid fungus on Kihansi Spray Toad population in the wild.

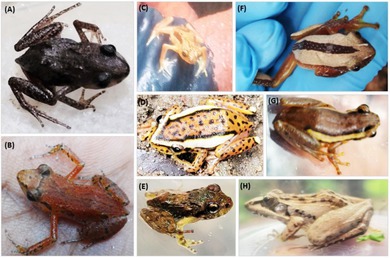

## 1 Introduction

The chytrid fungus, *Batrachochytrium dendrobatidis* (*Bd*), has caused declines and extinctions of amphibians across North America, Mesoamerica, Oceania, Europe, and Africa (Hanlon et al. 2018; Fisher et al. 2021). Originating from Asia, this pathogen has spread to other continents since the early 20th century, largely aided by the expansion of commercial trade of amphibians (Weldon et al. 2004; Hanlon et al. 2018). Notably, the pathogen is widespread in sub‐Saharan Africa from the west to the east coast and throughout southern African countries (Hopkins and Channing 2003; Weldon et al. 2004; Channing et al. 2006). In Africa, Ghose et al. (2023) documented an increase in *Bd* prevalence among 4623 amphibians from 3.2% between 1892 and 1999 to nearly 19% after the 2000s. A significant case of its devastating effect was observed in Tanzania, where *Bd* was detected on *Nectophrynoides asperiginis* in mid‐2003 (Weldon et al. 2020).

The *N. asperiginis*, an ovoviviparous amphibian, commonly known as the Kihansi spray toad (KST), is endemic to a 4700 m^2^ area of spray wetlands in the Kihansi Gorge Forest, within the Udzungwa Mountain ranges in southern‐central Tanzania. Its habitat was maintained by natural mists produced at the base of the Kihansi River waterfalls. In 1999, the commissioning of a dam for hydropower generation reduced the river water flow by 98% (Newmark 2002) causing desiccation of the spray wetlands and a decline in the KST's population by over 50%, from an estimated 20,163 individuals before the decline (Newmark 2002; Channing et al. 2006). Immediately, the Government of Tanzania, in collaboration with conservation partners, rescued the species by transferring around 500 toads to the Bronx and Toledo zoos in the United States in November 2000 to establish ex‐situ colonies, in case of extinction in their natural habitat. Simultaneously, it emulated the natural water spray wetlands by installing artificial mist irrigation systems (Newmark 2002; Channing et al. 2006).

The rescue measures were effective as the wild population, which had dwindled to less than 1500 individuals in mid‐2001 (Newmark 2002), rebounded to over 20,000 individuals by early June 2003 (Channing et al. 2006). However, this recovery was short‐lived because of a *Bd* outbreak, which led to a catastrophic decline of the *N. asperiginis* population in the wild by mid‐June 2003 (Channing et al. 2006; Weldon et al. 2020), from over 20,000 individuals recorded earlier in the year (Newmark 2002; Channing et al. 2006) to a handful of individuals. This rapid decline led ultimately to the species being declared extinct in the wild by 2009 (IUCN 2009). Subsequent studies revealed a widespread presence of the *Bd* pathogen in the Udzungwa Mountains. More recently, Sewell et al. (2024) identified the *Bd*‐CAPE lineage as the cause of KST extinction in the wild. The *Bd* pathogen was not present in the area before 2003, as confirmed by Weldon et al. (2020), who reported negative *Bd* tests through histopathology on 107 archived amphibian specimens, including *N. asperginis* and *N. tornieri* , collected between 1998 and 2002. This pathogen infects the skin of amphibians, causing epidermal hyperplasia and hyperkeratosis, which impedes respiration, thermoregulation, and exchange of electrolytes, often resulting in the host's death (Carver et al. 2010). However, some species are known to be more susceptible to *Bd* than others (Daszak et al. 2004; Carver et al. 2010).

The KST captive population in the USA, meanwhile, rose to over 5000 individuals by 2009 (Lee et al. 2006). This success enabled the establishment of two local captive breeding facilities in Tanzania at the University of Dar es Salaam and Kihansi and the initiation of a re‐introduction program by 2012 (Nahonyo et al. 2019). The initiation of the Kihansi facility, however, faced a challenge because of an outbreak of the *Bd* pathogen, identified as the Global Panzootic Lineage [*Bd*‐GPL] in 2013, but the spread was swiftly contained (Makange et al. 2014). A decade since the establishment of the captive breeding and re‐introduction program in Tanzania, about 24,000 toads have been released back into their natural habitat from these facilities, with 64% coming from Tanzanian centers and 36% from USA zoos (Ngalason et al. 2021). Despite these efforts, the survival and recruitment rates of the re‐introduced toads have been disappointingly low (Ngalason et al. 2019). Nevertheless, periodic releases of the growing captive KST population remain essential re‐introduction goals, fostering long‐term adaptation to natural habitats and managing space and resources sustainably in captivity.

The persistence of *Bd* in the Kihansi spray wetlands was further highlighted by a 6‐month pilot release experiment conducted in February 2022 to test whether post‐release mortality and poor adaptation of captive‐bred KSTs could partly reflect starvation, arising from difficulty in locating and consuming natural prey. In this experiment, 1000 captive‐bred individuals were released into the Midgorge Spray Wetland (MSW) in 10 separate aluminum wire mesh enclosures (92 cm × 62 cm × 62 cm), each housing 100 toads. The mesh allowed passive entry of local invertebrate prey from the surrounding environment (Figure S1). This experimental design was similar to that used by Mohamed and Magige (2020).

To assess the effect of food supplementation, toads in enclosures 1 through 9 (90%) received lab reared *Drosophila* spp. and *Collembola* on a tapering schedule designed to simulate a gradual transition to foraging independence: three times per week in month one, twice per week in month two, once per week in month 3, once every 2 weeks in month four, once in month 5, and none in month 6. Toads in enclosure 10 (10%) served as the control and received no supplemental lab food, but during the first 2 weeks, they were supported with live insects (*Afrosteles* sp.) trapped from the spray wetlands (Figure S2).

Toad numbers in each cage were observed and recorded on scheduled feeding days. Any dead toads observed were documented, collected, and preserved in clearly labeled jars containing 70% ethanol. The preserved specimens were stored at the Kihansi Station laboratory, pending funding for diagnostic testing to determine the cause of deaths. At the end of the experiment, a total census was conducted for each cage to assess survivorship.

Unfortunately, within 4 months, 93% of the toads—both lab food supplemented and non‐supplemented—had died; by 6 months, mortality had reached 98% across all enclosures. This pattern suggests that limited access to natural prey, and therefore starvation, was unlikely to be the primary driver of post‐release mortality. Despite the high losses, a notable outcome emerged: two toads born during the experiment survived long enough to produce both F1 and F2 generations. However, the F2 offspring did not survive, mainly because of a lack of resources for continued monitoring and support. This limited success offers hope, suggesting that in a well‐planned study, some individuals may eventually survive and reproduce, gradually increasing wild adaptation. These findings support continuing the release program. Moreover, as the captive (KST) population grows, strategic releases are necessary to prevent overcrowding, reduce disease risk, and maintain genetic diversity. Such releases also facilitate gradual exposure to *Bd*, promote long‐term persistence and enable the captive population to contribute to ecosystem restoration and broader conservation goals in the Kihansi Gorge.

However, before proceeding with additional KST releases, it is necessary to reassess the prevalence of *Bd*, which has been the primary hindrance to a successful re‐introduction program over the past 10 years (Weldon et al. 2020). *Bd* has persisted in the region for over 20 years (Weldon et al. 2020), likely because of the presence of co‐occurring amphibian species that act as reservoir hosts.

This study is therefore crucial for determining how to reduce *Bd*‐related mortality in KST and providing the Tanzanian Government with evidence‐based guidance on the most effective re‐introduction strategies. Even though it is very challenging to clean an environment contaminated by *Bd* to eradicate the fungus (Fisher et al. 2021), targeted conservation efforts might assist amphibian species in developing traits allowing them to coexist with the pathogen over time (McMahon et al. 2014; Knapp et al. 2016; Scheele et al. 2019).

Therefore, this study aims to evaluate the *Bd* status in the Kihansi Gorge spray wetlands by examining the KST individuals and other local amphibian populations. Specifically, we (i) examine the prevalence of *Bd* in three artificially misted spray wetlands; (ii) determine the distribution of the fungus in the spray wetlands; (iii) compare current *Bd* prevalence with levels documented in 2003/2006 (Weldon et al. 2020); examine whether (iv) the same *Bd*‐CAPE lineage reported by Sewell et al. (2024) is still circulating since 2003 in the Kihansi Gorge; and whether (v) *Bd* might have been responsible for the 98% deaths of the 1000 captive‐bred KST released into the wild on 28 February 2022. We hypothesize, on the basis of Fisher et al. (2021), that *Bd* continues to be prevalent in and affect the spray wetlands, impeding the successful re‐introduction and recovery of the KST population in the wild. We also anticipate finding the current *Bd* prevalence to be similar to the findings of Weldon et al. (2020), underscoring the persistent challenge of managing *Bd* in the Kihansi spray wetlands; and that *Bd* was the cause of massive deaths of KSTs 4 months after they were released into the wetlands in February 2022.

## 2 Materials and Methods

### 2.1 Study Site

**FIGURE 1:**
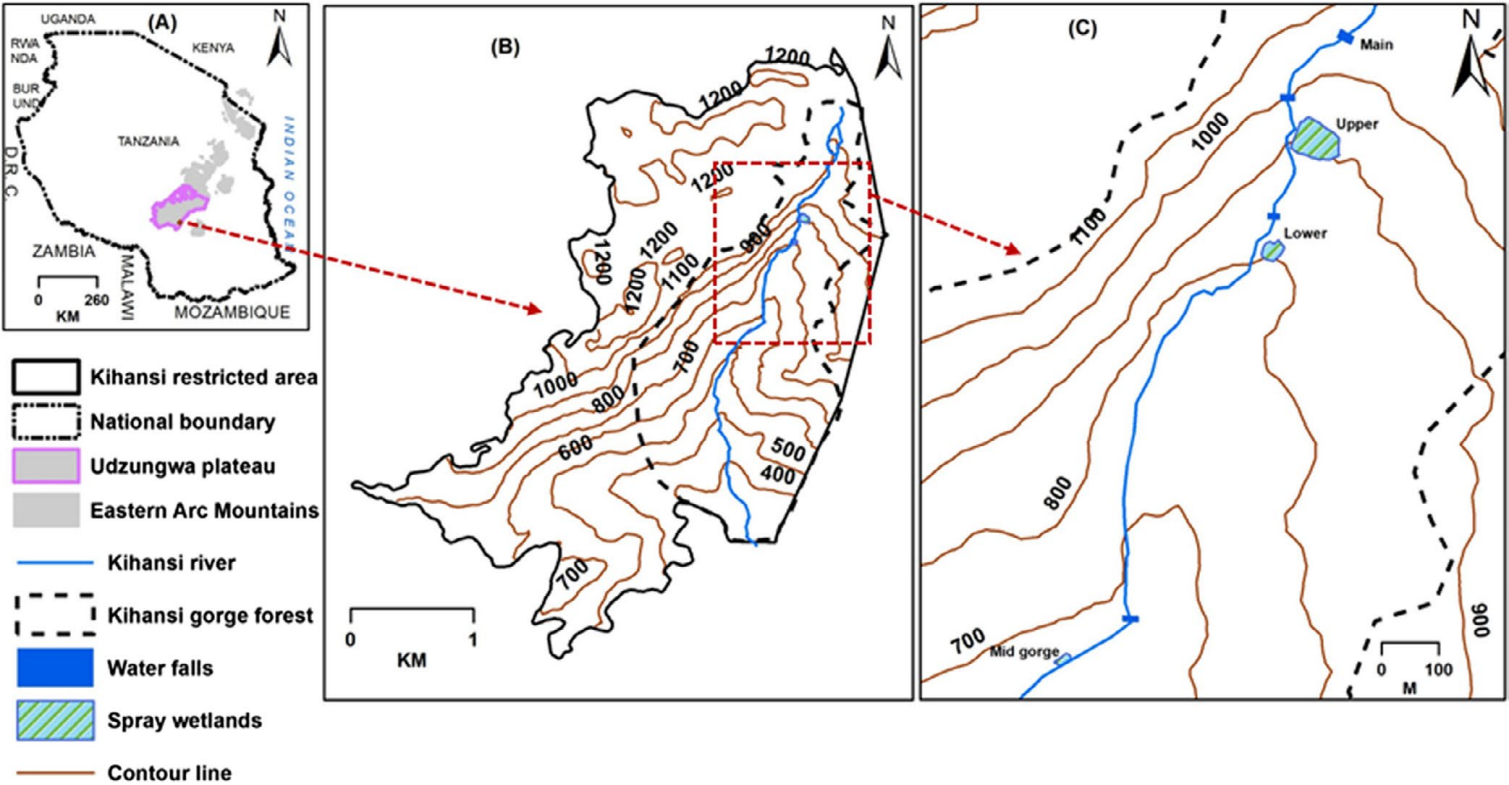
Location of the study site in Tanzania (A), the restricted area owned by the Tanzania Electric Supply Company (B), and the spray wetlands within the Kihansi Gorge Forest where the survey for chytrid fungus was conducted (C).

**FIGURE 2:**
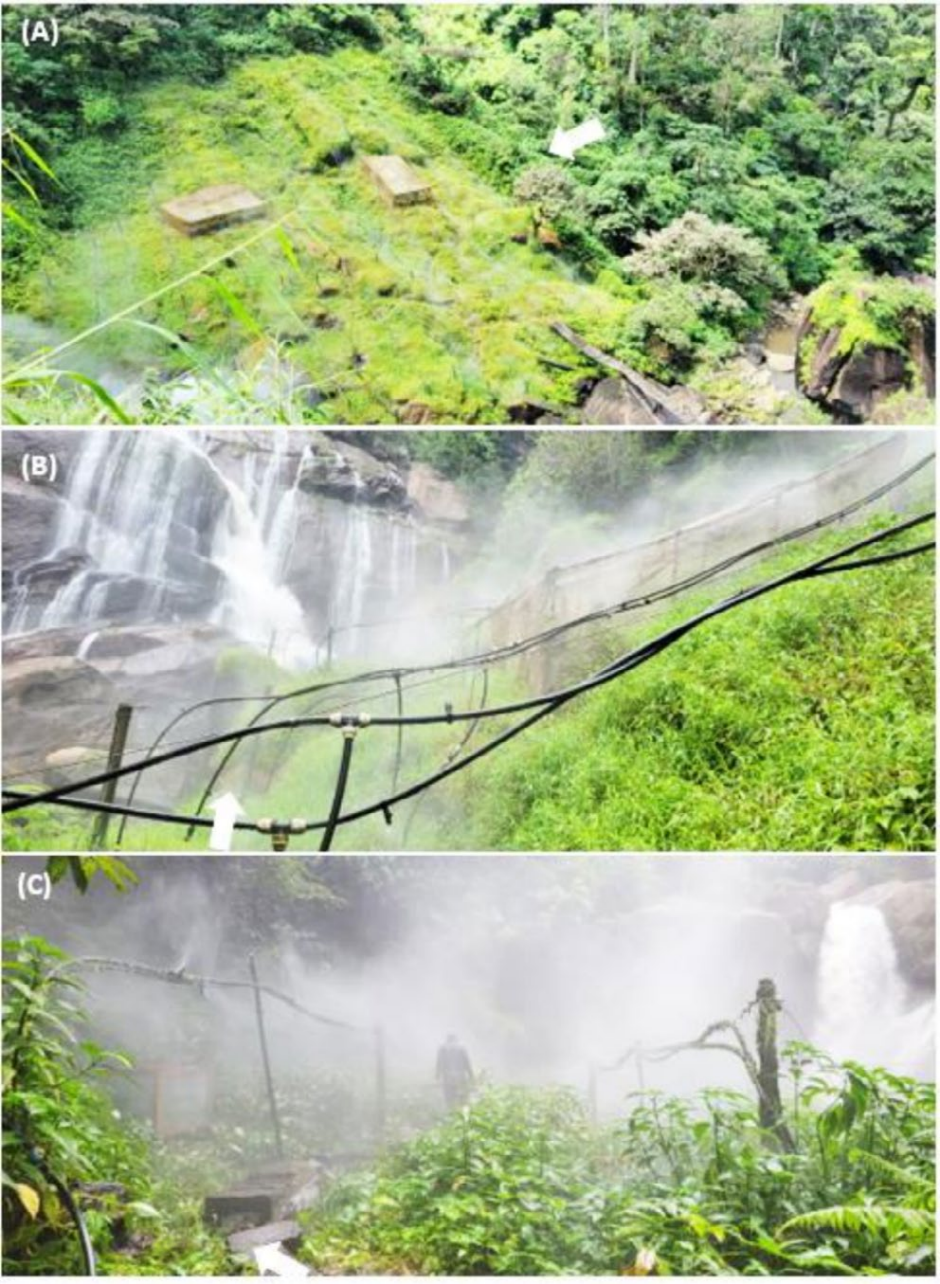
Three restored spray wetlands in Kihansi Gorge Forest in Tanzania: Upper spray wetland (USW) (A), lower spray wetland (LSW) (B), and mid Gorge spray wetland (MSW) (C), where the survey for *Batrachochytrium dendrobatidis* was conducted.

The KST inhabits the spray wetlands within the Kihansi Gorge Forest, located between latitudes 8.61° and 8.59° South, and longitudes 35.82° and 35.86° East, in south‐central Tanzania (Figure 1). This forest (20 km^2^) is part of the larger Udzungwa Mountain forests, renowned for their rich diversity of endemic flora and fauna, characteristic of the eastern arc mountain forests (Lovett et al. 1997; Poynton et al. 1998; Mtui et al. 2022). Before the diversion of the Kihansi River flow for hydropower generation, there was a series of five distinct spray wetlands in the gorge (Newmark 2002; Channing et al. 2006). Four of the five spray wetlands were maintained by Kihansi River water sprays generated at the foot of waterfalls (Figure 1). These comprised the main‐fall (1150 m elevation), upper (950 m), lower (750 m), and mid‐Gorge (635 m) spray wetlands (Figures 1 and 2). The fifth was Mhalala spray wetland (860 m) along the Mhalala stream, a tributary of the Kihansi River (Newmark 2002; Channing et al. 2006). The reduction of the Kihansi River flow, from an average of 16 to 2 m^3^/s, caused severe degradation of the spray wetlands (Newmark 2002; Channing et al. 2006).

In response, the four wetlands that derived water from the main‐stem of Kihansi River, except the main‐fall spray wetland, were subsequently restored (Figures 1 and 2) by installing artificial sprinkler systems, aiming to replicate the natural misting effect previously provided by the river. These systems continue to be maintained and monitored to ensure the survival of the KST population and the overall ecological health of the area, including the other floral and faunal species occurring in the wetlands. The restored spray wetlands now cover a total area of 4700 km^2^, distributed as 2900 m^2^ for the upper (USW), 1400 m^2^ for the lower (LSW), and 400 m^2^ for the mid‐Gorge (MSW) (Figure 2), supporting both the unique habitat requirements of the KST and the diverse species that share its environment.

The spray wetlands are dominated by short and dense herbaceous plants and low‐growing *Panicum* grasses and forbs including spike‐moss, *Selaginella kraussiana* (Kunze) A. Braun and fern, *Tectaria gemmifera* (Fée) Alston, *Impatiens*, *Streptocarpus*, *Begonia, Pilea*, *Alchemilla*, and *Brillantaisia* plants. These plants are frequently found in association with the KST, indicating their importance to the toad's habitat (Poynton et al. 1998). The spray wetland edges are dominated by *Afromomum mala* K. Schum and *Costus afer* Ker Gawl, adding to the biodiversity of the area. All the plant species inside and around the spray wetlands were common but declined after the desiccation caused by the reduced river flow. Plant species in the spray wetlands continue to recover after habitat restoration by artificial sprays (Poynton et al. 1998; Vandvik et al. 2014).

The spray wetlands are a habitat for a variety of amphibian species, including the KST, and the Southern torrent frog *Arthroleptides yakusini* Channing, Howell, and Moyer. In the vicinity of the wetlands, particularly in areas covered by sprays as well as in the surrounding forest, occurs Tornier's forest toad, *Nectophrynoides tornieri* (Roux) (Poynton et al. 1998; Channing et al. 2006). Other species less commonly reported in the area are: the Southern foam‐nest frog *Chiromantis xerampelina* Peters, 1854; Spotted reed frog *Hyperolius puncticulatus* (Pfeffer); and Fornasini's spiny reed frog *Afrixalus fornasini* (Bianconi) (Poynton et al. 1998; Channing et al. 2006).

After the detection of *Bd* in the spray wetlands in 2003, a hygienic protocol was established, where water baths containing 10% sodium hypochlorite were placed at the entrance to each spray wetland. Individuals entering or leaving the spray wetland are required to sterilize the sole of their shoes to minimize the spread of *Bd*.

The current microclimatic conditions within the spray wetlands have now been restored to closely resemble those before the diversion of the Kihansi River waters, with temperatures ranging from 15°C–20°C and relative humidity from 80% to 95% (Channing et al. 2006).

### 2.2 Data Collection

Surveys to sample amphibians were conducted both day and night, between 20–24 December 2022 for a total of 72 h of observations across the three spray wetland sites: 36 h at USW, 24 h at LSW, and 12 h at MSW. During the day, surveys followed systematic transects guided by sprinkler lines in the wetlands (Figure 2), whereas nighttime surveys were carried out opportunistically by safely following the walkways. The surveys involved searching for amphibians on rock surfaces as well as on top of and under vegetation. To avoid cross‐contamination between samples and between sampling sites, survey team members wore pairs of latex gloves, which were changed after handling each amphibian specimen, and cleaned their boots with sodium hypochlorite before proceeding to the next sampling location.

The amphibians collected during the surveys were kept in separate aerated plastic containers, each labeled with details specifying the area of collection (e.g., on rock surface, inside spray wetland, wetland edge). Animals were collected between 8 h and 2400 h, swabbed, and promptly released back into their original wetland within approximately 8 h of capture. To avoid sampling the same animal multiple times, the animals were temporarily held in containers located within the spray wetland, and care was taken to ensure that no animal was harmed.

#### 2.2.1 Swabbing Amphibians in the Field

We used fine‐tip dry swabs (MW113, Medical Wire & Equipment Co Ltd), supplied by Advantage Bunding, as recommended by Vredenburg and Briggs (Vredenburg and Briggs 2009). Two sets of skin‐swab samples were collected from individual animals, following a protocol developed by Vredenburg and Briggs (Vredenburg and Briggs 2009). Each skin‐swab sample contained three pieces of swabs collected from one adult individual: (i) the ventral surface of the animal (swabbed/scraped 30 times); (ii) the fore limbs, including armpits and toe‐webbings (each part swabbed 5 times), and (iii) the hind limbs, including pelvic area, thighs, and toe‐webbing (each part swabbed 5 times). For tadpoles, swabbing was restricted to the keratinized mouthparts, with both the upper and lower jaw sheaths and tooth rows swabbed (each part about 10 times) (Kadekaru and Une 2018). The three pieces of swabs were air‐dried for approximately 5 min in shaded, non‐humid conditions to minimize DNA degradation and prevent microbial growth or moisture that could inhibit the PCR reaction and were then preserved together in a labeled 3 ml cryovial. Each vial with 3 swabs was regarded as one sample.

The labels included institutional initials, sample number, and location to ensure accurate identification and tracking. The swab samples were preserved in a cooling box with icepacks within 10 h of collection in the field.

#### 2.2.2 Lab Preserved Specimens

To understand the cause of high mortality rates of KST during the pilot experiment, we selected 30 intact carcasses (whole‐body specimens) among specimens preserved at Kihansi laboratory. Those specimens were sourced from three identified preservation jars containing carcasses originating from enclosures number: 4 (sub‐adults), 8 (gravid), and 10 (mixed sub‐adults and juveniles). From each jar, 10 carcasses were selected and subdivided into two 15 ml cryovials, each containing five animals. In total, six cryovials were prepared, each filled with 70% ethanol containing five carcasses. This procedure resulted in three sets of laboratory‐preserved carcass samples prepared for subsequent analyses.

The field skin‐swab samples and lab‐preserved sets of samples were combined to form two identical batches of 44 samples for shipment to the laboratory for *Bd* analysis. Each batch contained one set of 41 field skin‐swab samples (three swab pieces per sample) and one set of 3 preserved whole‐animal samples (five carcasses per sample), totaling 123 swab pieces and 15 carcasses per batch. One batch was sent to Sokoine University of Agriculture's SACIDS Molecular Biology Laboratory in Tanzania, and the other batch was shipped to San Diego Zoo Wildlife Alliance's amphibian diseases laboratory in the United States of America.

All individuals encountered in the field were photographed and identified to species level using Harper's field guide (Harper et al. 2010). Digital photos of unknown or cryptic individuals were sent through WhatsApp to herpetologists at the Universities of Dar es Salaam (Tanzania) and Manchester Metropolitan (Italy) for prompt identification. Because of the small sizes of the surveyed wetlands, locations of individuals detected with chytrid fungus were plotted on the basis of photos of the spray wetlands using color codes to illustrate the detailed distribution of fungus.

Baseline data on prevalence of amphibian species documented in the spray wetlands in 2003 and 2006 were obtained from Weldon et al. (2020) for comparison with the current status of *Bd* in the area. We used a similar approach and effort in surveying amphibians in the Kihansi spray wetlands as Weldon et al. (2020), to ensure that our findings could be directly compared with theirs.

The field data collections were performed in compliance with ARRIVE guidelines (Percie du Sert et al. 2020), the research guidelines and regulations set by the Tanzania Wildlife Research Institute (TAWIRI) in August 2020 and after ethical review by TAWIRI's ethical review committee, and after acquiring a research permit no. 2020435NA2018259 from the Tanzania Commission for Science, Technology, and Innovation (COSTECH). This ensured that all research activities adhered to the required legal and ethical standards.

### 2.3 Detection and Quantification of Batrachochytrium *Dendrobatidis*

Molecular detection of *Bd on all field‐collected and lab‐preserved amphibian samples was performed* using conventional PCR (cPCR) (Annis et al. 2004) at Sokoine University of Agriculture and quantitative PCR (qPCR) (Hyatt et al. 2007) at San Diego Zoo Wildlife Alliance, followed by cPCR and nucleotide sequencing of positive samples. Our intention in conducting the PCR analysis was not to obtain quantitative values. Instead, we performed qPCR to leverage the assay's high sensitivity and specificity for the qualitative detection of *Bd* (presence/absence).

#### 2.3.1 Conventional PCR Procedures

Conventional PCR procedures prior to DNA extraction, the three samples containing whole body lab‐preserved specimens were rinsed in phosphate‐buffered saline (PBS) to remove ethanol residues. Then skin tissues were scraped from the toe webs with one sterile toothpick per sample as described by Annis et al. (2004), forming three skin‐tissue scrapings in pooled samples. DNA was then extracted from a total of 44 samples, including 41 field‐skin‐swab and 3 lab‐preserved skin tissue pooled samples using Qiagen Blood and Tissue extraction kit (Qiagen, Hilden, Germany) following the manufacturer's instructions. The molecular detection of *Bd* from the recovered DNA was achieved using Eppendorf Mastercycler nexus PCR thermal cycler (Eppendorf, Hamburg, Germany), targeting the fungal 5.8S rRNA gene and flanking internal transcribed spacer 1 (ITS1) and ITS2 regions. Primers Bd1a (5′‐CAGTGTGCCATATGTCACG‐3′) and Bd2a (5′‐CATGGTTCATATCTGTCCAG‐3′) were employed as previously described by Annis et al. (2004). Each 25 μL volume PCR reaction contained 2× Taq enzyme mix (12.5 μL), RNase‐free water (7.5 μL), 10 μM of forward primer (1 μL), 10 μM of reverse primer (1 μL), and DNA sample (3 μL). The PCR conditions consisted of initial denaturation at 95°C for 10 min followed by 40 cycles of denaturation (45 s at 95°C), annealing (45 s at 60°C), and extension (1 min at 72°C), and a final extension at 72°C for 10 min. PCR product validation was conducted via electrophoresis performed at 100 V and 200 mA for 30 min using a 1.5% agarose gel mixed with Gel Red nucleic acid stain (Phenix Research Products) and a 6× loading dye (Promega, Wisconsin, USA). A BioDoc‐It imaging system (Bio‐Rad, Hercules, CA, USA) was used for visualization and confirmation of the amplified *Bd* PCR products.

#### 2.3.2 Quantitative PCR Procedures

Quantitative PCR procedures before conducting DNA extraction, the whole‐body lab specimens preserved in alcohol were rinsed two times in PBS. Each sample of 5 individual carcasses per tube was swabbed with one swab. There were three tubes (samples containing 5 carcasses each) in total, creating three pooled skin‐swab samples. DNA was then extracted from 44 skin‐swab samples (i.e., three pooled skin‐swabs from lab preserved samples and 41 skin swabs collected in the field) using the Qiagen DNeasy Blood and tissue kit (Qiagen, Hilden, Germany) as per manufacturer's protocol. Molecular detection of *Bd* was performed using the Taqman qPCR Bd assay as described in Hyatt et al. (2007) and Boyle et al. (2004), with primers (ITS1‐3 Chytr 5′‐CCTTGATATAATACAGTGTGCCATATGTC‐3′ and 5.8S Chytr 5′‐AGCCAAGAGATCCGTTGTCAAA‐3′) and probe (Chytr MGB2 5'6FAM‐CGAGTCGAACAAAAT‐MGBNFQ‐3′) designed to target the ITS1 rRNA gene of *Bd*. Samples were run in triplicate on a Quantstudio 6 flex (ThermoFisher Scientific, Carlsbad, CA, USA) using 384‐well plates. An Exogenous Internal Positive Control (EIC) (Thermo Fisher Scientific) was included for each sample to detect PCR inhibition. Commercially available serial diluted plasmid standards (Pisces Molecular LLC, Boulder, CO, USA), along with negative controls, were included for calibration and validation. Each qPCR reaction consisted of 2× Environmental Taqman Mastermix 2.0 (Thermo Scientific) (10 μL), 100 μM forward Bd primer (0.18 μL), 100 μM reverse Bd primer (0.18 μL), 0.25 μM chytrid probe Chytr MGB2 (0.05 μL), 20 mg/μL bovine serum albumin (BSA) (0.2 μL), DNA template (5 μL), and nuclease‐free water (4.39 μL), making a total volume of 20 μL. The qPCR utilized the Taqman program consisting of 1 cycle of 56°C for 2 min followed by 95°C for 10 min, then 55 cycles at 95°C for 15 s and 60°C for 1 min. The outcome was determined on the basis of cycle threshold (Ct) values. Samples amplifying at Ct ≥ 50 were considered not detected, those amplifying at Ct < 50 in at least two wells were deemed positive, and those amplifying in only one well of three were subject to a rerun in five wells with an extra one for EIC. Samples that amplified with a Ct < 50 in at least 3 wells were considered positive (Table S2). The sequences generated were submitted to the National Center for Biotechnology Information (NCBI) GenBank and assigned accession numbers PP188411‐13.

### 2.4 Detection of *Batrachochytrium dendrobatidis* Strain CAPE

Following the procedure described in (Ghosh et al. 2021), samples that tested qPCR positive for Bd were then analyzed using a lineage‐specific qPCR diagnostic for Bd CAPE to confirm whether the type of Bd strain occurring in the Kihansi Gorge was Bd CAPE, as reported by Sewell et al. (2024). This requirement came later after Sewell et al. (2024) had suggested that the extinction of KST in 2003 was caused by Bd‐CAPE.

### 2.5 Statistical Analysis

We compared our 2022 Bd prevalence results with those reported by Weldon et al. (2020). First, we obtained the 2000s amphibian secondary datasets (combined 2003 and 2006) from Weldon et al. (2020) and, using R software, we calculated *Bd* prevalence, expressed as the proportion of infected individuals with 95% Wilson binomial confidence intervals (Wilson 1927; Zou and Donner 2008). We then used a two‐sample test for equality of proportions to assess differences in *Bd* prevalence (proportion infected) among the seven amphibian species examined for pathogen infection in both the 2000s and 2022. We used logistic regression to test whether the 2022 *Bd* prevalence values for all animals that tested positive (response variable) were influenced by location (i.e., spray wetland), species, or the interaction between species and location (independent variables) among all animals tested for chytrid fungus that year. Only qPCR *Bd* results were considered for analysis. Three separate logistic models were fitted using the GLIMMIX procedure in the SAS software (SAS Institute Inc 2021), with a binary response variable (presence/absence of chytrid fungus): a model with (1) three location levels (USW, LSW, and MSW) and (2) seven species, and the interaction of species and location as predictor variables. Although individuals of each species were classified as adults or juveniles, the life stage was excluded from statistical analysis because of the small sample size. Unless stated otherwise, statistical significance was assessed at alpha = 0.05. Only data generated using qPCR were used in the statistical analyses because of their higher reliability.

## 3 Results

### 3.1 Species Composition and Sample Size Per Spray Wetland

**FIGURE 3:**
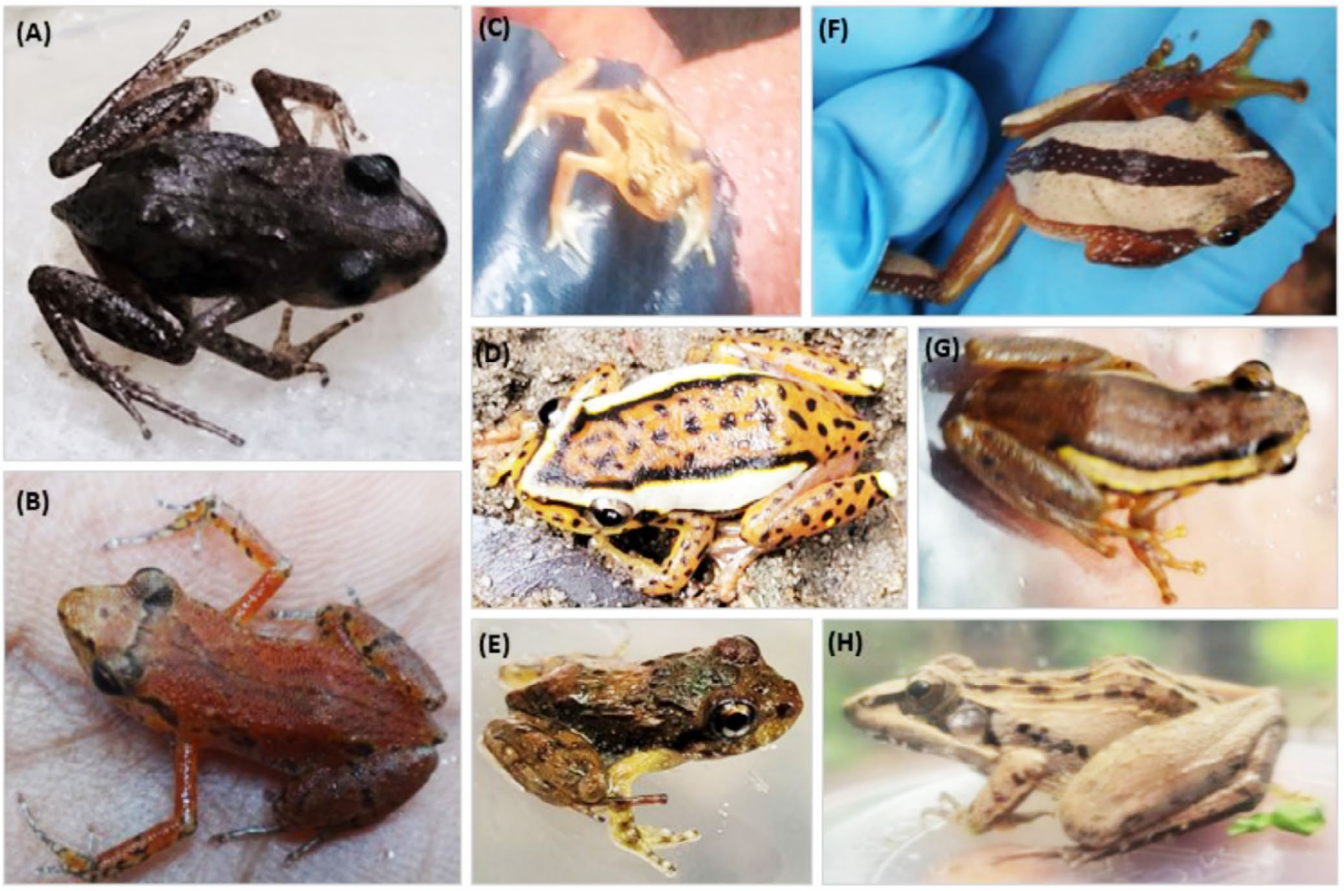
Species of amphibians sampled at the upper, lower, and mid‐Gorge spray wetlands: *Arthroleptis xenodactyloides* (A and B), *Nectophrynoides asperginis* (C), *Hyperolius mitchelli* (D), *Arthroleptides yakusini* (E), *Afrixalus fornasinii* (F), *Hyperolius substriatus* (G), and *Ptychadena anchietae* (H).

A total of seven amphibian species, including *Afrixalus fornasinii* (Bianconi), *Arthroleptides yakusini* Channing, Howell, and Moyer, *Arthroleptis xenodactyloides* Hewitt, *Hyperolius mitchelli* Loveridge, *H. substriatus* Ahl, *Ptychadena anchietae* (Bocage), and *Nectophrynoides asperginis* Poynton, Howell, Clarke & Lovett, were sampled in the spray wetlands (Table 1 and Figure 3). *Arthroleptides yakusini* was the most common and abundant species across the wetlands, and all age classes (adults, juveniles, tadpoles) were observed (Table 1). From those seven species, we obtained a total of 44 samples composed of adult individuals (88.7%), juveniles (4.5%), and tadpoles (6.9%) (Table 1). Of the samples, 89% were collected during the night surveys (Table S1), and the remaining were either captured during daytime surveys (4%) or were from lab‐preserved specimens (7%) (Table S1).

**TABLE 1:** Relative proportion of samples of amphibian species collected from the spray wetlands (upper (USW), lower (LSW), and mid‐Gorge (MSW) in the Kihansi Gorge Forest in Tanzania). Numbers of samples per species are in parentheses.

Species name	USW	LSW	MSW	Total	Age composition
*Afrixalus fornasinii*		2% (1)	2% (1)	5% (2)	Adults
*Arthroleptids yakusini*	14% (6)	25% (11)	11% (5)	50% (22)	18 adults, 1 juvenile, and 3 tadpoles
*Arthroleptis xenodactyloides*	2% (1)	2% (1)		5% (2)	Adults
*Hyperolius mitchelli*	2% (1)			2% (1)	Adults
*Hyperolius substriatus*	16% (7)			16% (7)	Adults
*Nectophrynoides asperginis* ^a^			11% (5)	11% (5)	4 adults, 1 juvenile
*Pytchadena anchietae*	5% (2)	7% (3)		11% (5)	Adults
Total	39% (17)	36% (16)	25% (11)	100% (44)	

^a^ KST samples collected for analysis. These included five KSTs, of which two samples were obtained directly from the field, and three were lab‐preserved, originating from a pilot experiment.

### 3.2 Prevalence and Distribution of *Batrachochytrium dendrobatidis* in the Kihansi Gorge Spray Wetlands

**FIGURE 4:**
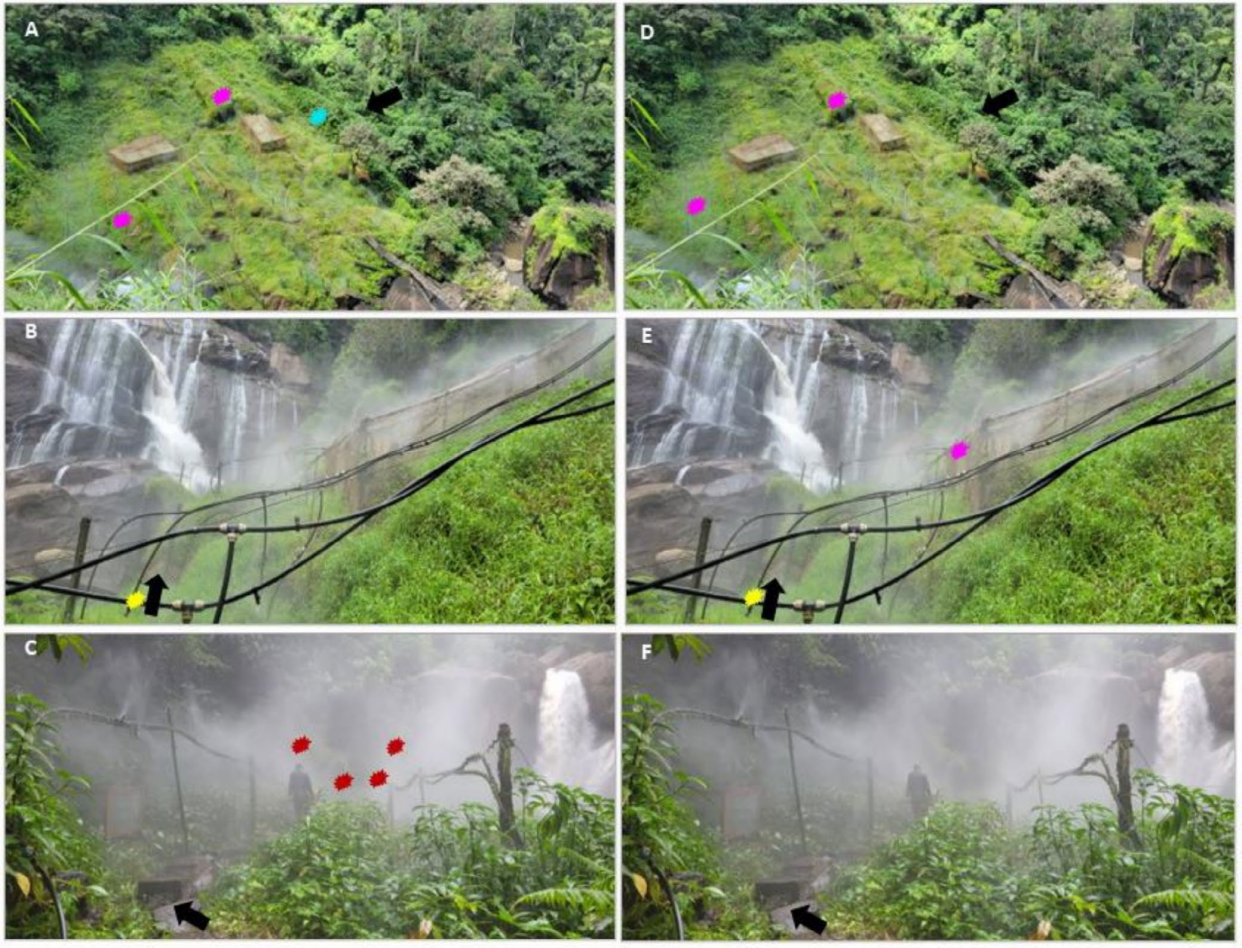
Distribution of *Batrachochytrium dendrobatidis* (*Bd*) across the upper (A, D), lower (B, E), and mid‐Gorge (C, F) spray wetlands. The red, pink, yellow, and aqua‐colored symbols show the locations where *Bd* infected amphibians — *Nectophrynoides asperginis* , *Arthroleptides yakusini* , *Pytchadena anchietae*, and *Hyperolius substriatus* —were collected, respectively. Panels ABC (left) and DEF (right) show samples that tested positive for *Bd* through quantitative and conventional Polymerase Chain Reaction methods, respectively. The black arrows show the entrance to the wetlands.

Overall, *Bd* was detected in 32% of the 44 samples across the three spray wetlands, and pathogen prevalence per wetland ranged from 19% to 55% (Table 2). The *Bd* infection was specifically detected in four out of seven species: *A. yakusini* , *N. asperginis* , *P. anchietae,* and *H. substriatus* (Figure 4), with pathogen prevalence ranging from 6% to 36% per species per wetland (Table 2). Four out of 5 specimens of *N. asperginis* , which were composed of three adults and one juvenile, all lab‐preserved specimens, tested positive for *Bd* (Table 2 and Table S1), whereas only adults of *A. yakusini* , *P. anchietae,* and *H. substriatus* were infected. The fifth *N. asperginis* specimen that tested negative was collected alive on vegetation leaves outside an enclosure (Table S1). *Bd* prevalence was not significantly associated with species, wetland, or their interaction ($F_{3,76}=0,P>0.05$, Table S3).

**TABLE 2:** Prevalence of *Batrachochytrium dendrobatidis* across spray wetlands and among species in the Kihansi Gorge, Tanzania.

Site	Scientific name of species	Life stage	Total # samples (# of samples with infection)	Prevalence (95% CI)
Upper spray wetland	*Arthroleptids yakusini*	Adult	6 (3)	0.18 (0.06–0.41)
	*Arthroleptis xenodactyloides*	Adults	1 (0)	
	*Hyperolius mitchelli*	Adults	1 (0)	
	*Hyperolius substriatus*	Adults	7 (1)	0.06 (0.01–0.27)
	*Ptychadena anchietae*	Adults	2 (1)	0.06 (0.01–0.27)
	Sub‐total		17 (5)	0.29 (0.13–0.53)
Lower spray wetland	*Afrixalus fornasinii*	Adult	1 (0)	
	*Arthroleptides yakusini*	Adult	11 (1)	0.06 (0.01–0.28)
	*Arthroleptis xenodactyloides*	Adult	1 (0)	
	*Pytchadena anchietae*	Adults	3 (2)	0.13 (0.03–0.36)
	Sub‐total		16 (3)	0.19 (0.07–0.43)
Mid‐gorge spray wetland	*Afrixalus fornasinii*	Adult	1 (0)	
	*Arthroleptides yakusini*	Adults	5 (2)	0.18 (0.05–0.48)
	*Nectophrynoides asperginis*	Three adults, one sub‐adult	5 (4)	0.36 (0.15–0.65)
	Sub‐total		11 (6)	0.55 (0.28–0.79)
	Total samples		44 (14)	0.32 (0.20–0.47)

### 3.3 Confirming the Presence of *Batrachochytrium dendrobatidis* CAPE

Overall, nearly 70% of the *Bd* positive samples (10 out of 14) spanning four species ( *A. yakusini* , *H. substriatus*, *P. anchietae* , and *N. asperginis* ), across the three spray wetlands belonged to *Bd*‐CAPE lineage (Table 3); the remaining 30% were undetermined.

**TABLE 3:** Confirmation of *Batrachochytrium dendrobatidis* CAPE result (Ct stands for Cycle threshold, a measure of Positive Quantitative Polymerase Chain Reaction Product; SD means standard deviation).

Sample ID	*Bd*‐host name	Life stage	*Bd‐*CAPE Ct	*Bd‐*CAPE Ct	CAPE Ct mean	CAPE Ct SD	*Bd‐*CAPE qPCR result
TWS0045	*Nectophrynoides asperginis*	Adult	22.367	22.178	22.272	0.133	Positive
TWS0037	*Nectophrynoides asperginis*	Adult	24.117	24.788	24.453	0.475	Positive
TWS0046	*Nectophrynoides asperginis*	Adult	23.569	26.369	26.327	0.06	Positive
TWS0047	*Nectophrynoides asperginis*	Adult	26.384	26.484	26.436	0.067	Positive
TWS0013	*Pytchadena anchietae*	Adult	27.481	27.858	27.669	0.267	Positive
TWS0020	*Hyperolius substriatus*	Adult	31.709	31.627	31.668	0.058	Positive
TWS0035	*Arthroleptides yakusini*	Adult	34.132	33.789	33.96	0.242	Positive
TWS0032	*Arthroleptides yakusini*	Adult	36.572	36.039	36.306	0.377	Positive
TWS0031	*Arthroleptides yakusini*	Adult	37.555	36.349	36.952	0.853	Positive
TWS0040	*Arthroleptides yakusini*	Adult	38.278	38.111	38.195	0.118	Positive
TWS0042	*Arthroleptides yakusini*	Adult	Undetermined	38.65	38.265	NA	Positive
TWS0014	*Pytchadena anchietae*	Adult	Undetermined	49.31	49.31	NA	Not detected
TWS0033	*Pytchadena anchietae*	Adult	Undetermined	Undetermined	NA	NA	Not detected
TWS001	*Arthroleptides yakusini*	Adult	Undetermined	Undetermined	NA	NA	Not detected

### 3.4 Distribution of *Batrachochytrium dendrobatidis* in the Kihansi Gorge Spray Wetlands

*Batrachochytrium dendrobatidis* was detected inside and at the edges and entrances to the spray wetlands (Figure 4) by both the qPCR and cPCR detection methods (Table 4), except for the MSW, where the pathogen was detected by qPCR only (Table 4 and Figure 4). Of the four samples of *N. asperiginis* detected with *Bd*, three were lab‐preserved specimens originally collected in the cage in the MSW during the pilot study, and one was from an F1 generation sub‐adult found dead in the experimental cage in the MSW during the field survey (Table S1). The cage also housed an F1 gravid female. Note that during data collection, we chose not to swab the remaining gravid female to avoid any stress that could potentially lead to abortion. The only individual sample of *N. asperiginis* that tested negative for *Bd* was collected from the top of vegetation, possibly an escapee from experimental cages (Table S1). The individuals of *A. yakusini* detected with the pathogen at USW and LSW were collected from rock surfaces inside the spray wetland, whereas the infected *P. anchietae* and *H. substriatus* individuals were found on vegetation at the entrance to the spray wetlands (Table 4 and Table S1).

**TABLE 4:** Detection of *Batrachochytrium dendrobatidis* (*Bd*) by quantitative and conventional polymerase chain reaction from samples collected from the spray wetlands [upper (USW), lower (LSW), and mid‐Gorge (MSW) in Kihansi Gorge, Tanzania]. *Nectophrynoides asperginis* : 5 skin‐swabs were analyzed using quantitative polymerase chain reaction (qPCR); 2 skin‐swabs and 3 tissues were analyzed using conventional polymerase chain reaction (cPCR). The numbers at the end of each bar are samples infected with *Bd*.

Species name	Samples examined		*Bd* detected (%/#) at USW		*Bd* detected (%/#) at LSW		*Bd* detected (%/#) at MSW		Total	
	No.	Type	qPCR	cPCR	qPCR	cPCR	qPCR	cPCR	qPCR	cPCR
*Afrixalus fornasinii*	2	Swab			0%	0%	0%	0%	0%	0%
*Arthroleptids yakusini*	22	Swab	14% (3)	9% (2)		4.5% (1)	9% (2)		23% (5)	14% (3)
*Arthroleptis xenodactyloides*	2	Swab	0%	0%	0%	0%			0%	0%
*Hyperolius mitchelli*	1	Swab	0%	0%					0%	0%
*Hyperolius substriatus*	7	Swab	14% (1)	0%					14.3% (1)	0%
*Nectophrynoides asperginis*	5	Swab/tissue					50% (4)	0%	50% (4)	0%
*Pytchadena anchietae*	5	Swab	20% (1)		40% (2)	40% (2)			60% (3)	40% (2)
Total	44		11.4% (5)	4.5% (2)	4.5% (2)	7% (3)	14% (6)	0% (4)	29.5% (13)	11% (5)

### 3.5 Comparing the Current and Historical Levels of *Batrachochytrium dendrobatidis* Prevalence in the Kihansi Spray Wetlands

**FIGURE 5:**
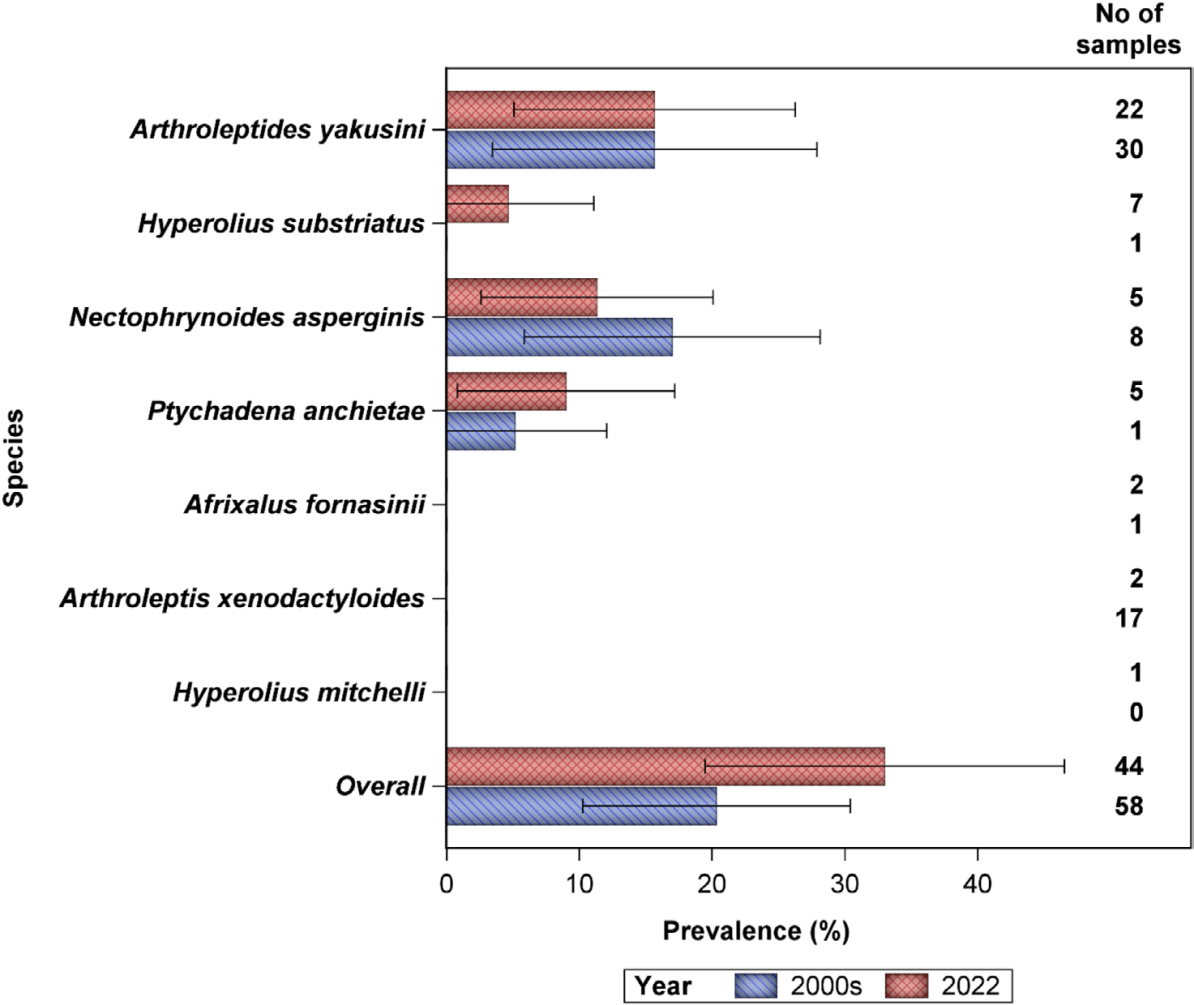
Comparison of *Batrachochytrium dendrobatidis* prevalence levels in the Kihansi Gorge spray wetlands between the 2000s and 2022.

We documented seven species of amphibians (Figure 5) which were also documented by Weldon et al. (2020) to be present in the Kihansi spray wetlands (Figure 5). In addition to three species that were reported with *Bd* infection in the past 20 years (Weldon et al. 2020), we report *Bd* infection of *H. substriatus,* which was not reported before by Weldon et al. (2020). The *Bd's* level of prevalence reported in the 2000s (Weldon et al. 2020) was not significantly different from those found in this study [*t*‐test: Mean estimates for 2000s = 0.044 and for 2022 = 0.046 (95% confidence interval = −0.073–0.069); *t* = −0.065, df = 12, *p* = 0.949].

## 4 Discussion

We assessed the prevalence and distribution of the *Batrachochytrium dendrobatidis* (Bd) within three spray wetlands covering a total of 4700 m^2^ (Newmark 2002). We acknowledge the small amphibian population size within the Kihansi Gorge spray wetlands, a limitation also noted by Weldon et al. (2020), who in 2003 and 2006 confirmed that chytrid fungus was the proximate cause of the Kihansi spray toad's extinction. Our findings, therefore, provide essential new data and insights for ongoing conservation efforts to re‐introduce the Kihansi spray toad into its native habitat.

Overall, our findings show that *Bd* remains prevalent across the Kihansi spray wetlands, confirming continuity in the presence of *Bd* infection among amphibian species identical to those reported two decades ago (Weldon et al. 2020). Consistent with Sewell et al. (2024), we confirmed the presence of *Bd*‐CAPE in the Kihansi gorge and found that this *Bd* variant likely caused the 98% mortality of the 1000 KSTs released into the MSW in February 2022, 4 months prior to our study. In addition to the species previously identified by Weldon et al. (2020) as *Bd*‐positive in the same locality— *N. asperginis* (Bufonidae), *A. yakusini* (Ranidae), and *P. anchietae* (Ptychadenidae)—we identified *H. substriatus* (Hyperoliidae) as the third likely *Bd* carrier. The prevalence levels of *Bd* in the latter three species are similar to those reported previously, underscoring the pathogen's ongoing impact.

Similar to Weldon et al. (2020), our findings show that *N. asperginis* remains the most vulnerable to *Bd*, whereas the other three sympatric species, although also testing positive for *Bd* infection, do not show signs of disease and are likely carriers only. For example, *A. yakusini* continues to breed and has remained common and abundant in the area, compared to the other species, indicating the resilience of certain species to *Bd* infection. This resilience is reflected in broader surveys across the Udzungwa Mountain forests and Kilombero valleys in Tanzania, where *Bd* infection was noted without signs of sickness in five amphibian species or genera (Sewell et al. 2024).

In contrast to previous research (Moyer and Weldon ; Sewell et al. 2024) that reported all 48 samples of *Ptychadena* as *Bd*‐negative, we found positive *Bd* in three out of five samples of the same genus, similar to Weldon et al. (2020) who detected *Bd* in one *Ptychadena* sample.

The vulnerability of *N. asperginis* and the resistance of sympatric species to *Bd* have been observed and documented in amphibians globally. For example, in the United States of America, poison dart frogs, *Dendrobates tinctorius* (Cuvier), died of severe chytridiomycosis, whereas bullfrogs, *Rana catesbeiana* (Shaw), in the same locations, were resistant to the disease (Daszak et al. 2004). Similarly, in the Ethiopian highlands and the Albertine Rift Valley in Africa, a significant number of amphibian species have been identified as *Bd* carriers (Gower et al. 2012; Seimon et al. 2015). This marked variability in susceptibility and resistance highlights the complex interactions between *Bd* and its amphibian hosts. The diversity in host‐pathogen dynamics points to the importance of considering species‐specific and environmental factors in assessing the threat *Bd* poses to amphibian biodiversity.

Variations in *Bd* prevalence may reflect local weather conditions at Kihansi, which are more favorable to *Bd* than in other parts of the Udzungwa Mountains. Generally, *Bd* prevalence is higher in amphibians inhabiting cooler (below 23°C) and wetter environments (Kriger et al. 2007; Scheele et al. 2015; Verster et al. 2024) mirroring the conditions in the Kihansi Gorge spray wetlands. These conditions are also known to influence the occurrence and co‐occurrence of different *Bd* types in a single location or host (Verster et al. 2024). For example, the *Bd*‐GPL lineage generally occurs in drier and warmer sites at lower elevations, whereas *Bd*‐CAPE is found in areas with high precipitation and low temperature at higher elevations, but there are also instances where the two strains overlap at the highest elevations (Verster et al. 2024). The co‐occurrence of multiple *Bd* types in one location has been documented (Miller et al. 2018; Byrne et al. 2019; Doherty‐Bone et al. 2020) and is widely associated with mass mortality and extinctions of amphibians (Byrne et al. 2019), as reported in Cameroon (Miller et al. 2018; Byrne et al. 2019; Doherty‐Bone et al. 2020) and South Africa (Ghosh et al. 2021). Because of limited resources, we did not test *Bd strains* other than *Bd*‐CAPE. Consequently, it remains unclear whether multiple *Bd* strains co‐occur in the Kihansi Gorge, especially since some species known to co‐exist with *Bd*‐CAPE in other parts of the Udzungwa Mountains repeatedly tested negative for *Bd* in the Kihansi Gorge, as observed in this study and by Weldon et al. (2020). Furthermore, sympatric species, such as *A. yakusini* and *Ptychadena* sp., although living alongside *Bd*‐CAPE in the Kihansi Gorge, were reported as *Bd*‐negative in the Udzungwa Mountains by Sewell et al. (2024). These findings suggest that *Bd*‐CAPE is lethal to *N. asperginis* but not to other sympatric species in Kihansi Gorge, which may act as carriers or resistant hosts, implying the possible involvement of an unidentified factor that amplifies *Bd*'s lethality specifically in *N. asperginis* .

The detection of *Bd* in this study was achieved using cPCR and qPCR methods, with both approaches yielding similar results. Nevertheless, the qPCR method appeared to be more sensitive in detecting *Bd* in *N. asperginis* than cPCR. The enhanced sensitivity and reliability of qPCR over other methods have also been previously reported (Boyle et al. 2004; Kriger et al. 2006; Hyatt et al. 2007). Although the primary aim of our research was not to assess the comparative efficacy of these methods, the consistent and reliable results provided by both methods are crucial for informed decision‐making regarding the presence of *Bd* in the studied amphibian populations.

The findings from both *Bd* detection methods enable us to confidently advise the government and conservation managers in the United Republic of Tanzania of the persistent presence of the chytrid fungus within the Kihansi Gorge. This underscores the crucial need for continued, effective human intervention and adequate financial commitment to ensure the success of the KST re‐introduction program. Fischer et al. (2021) stress that it is very unlikely that an environment already contaminated with the *Bd* pathogen can be completely cleaned up. However, targeted human interventions such as active management strategies to mitigate concurrent threats (Scheele et al. 2019) and pre‐exposing individuals in captive breeding programs to low‐virulence strains of fungi before their release can enhance adaptation and tolerance to *Bd* infections in amphibian populations (McMahon et al. 2014). The offspring born in an environment with *Bd* may acquire immunity against *Bd*, hence establish a population that can survive with *Bd*.

Previous studies (Voyles et al. 2018) have shown that amphibian species raised in captivity exhibit a reduced capacity to resist *Bd* infections compared to their wild counterparts. Our findings reinforce this, as spray toads released from captivity during the pilot study struggled to survive in the wild because of lower resilience to the *Bd* pathogen compared to their offspring (F1) born in the wild, which survived long enough to give birth to F2 offspring. Specifically, the *N. asperginis* individuals that were found dead and *Bd*‐positive during this study had been bred in captivity and later released into the wetlands.

Although our field monitoring experiment aimed to mimic the process of aiding KST adaptation and recovery in a *Bd*‐infected environment, similar to strategies used for *R. sierrae* (Knapp et al. 2016), the survival rate at the end of the experiment was a dismal 2.2%. During the 6‐month period, 31 babies were born within the cages, yet only two—one male and one female—survived to maturity, yielding a survival rate of merely 0.06%. In January 2023, the surviving female gave birth to 5 babies (F2 generation), but only one survived until the end of April 2023 (Figure S2). These results highlight the significant challenges faced in promoting survival and reproduction among captive‐bred toads re‐introduced to their natural, albeit infected, habitats.

Given the low likelihood of eradicating the fungus from the spray wetlands, we advocate for the continuation of experimental releases of captively reared toads, alongside diligent monitoring and management of concurrent threats. This includes ensuring sufficient water sprays into the wetlands and strict adherence to the established hygienic protocols for anyone entering and leaving the wetlands. Such measures aim to facilitate a scenario in which the KST can co‐exist with the *Bd* pathogen within its natural habitat. Over time, these carefully managed releases may support the persistence of a population capable of withstanding *Bd* exposure, contributing to the long‐term conservation of the species, mirroring the success observed with *R. sierrae* (Knapp et al. 2016). This approach underscores a long‐term strategy for conservation, focusing on resilience and adaptation within the KST population to secure their future in the wild.

Since the *Bd* is widespread across the Udzungwa Mountains and probably in the whole of the eastern arc forest mountains, the successful re‐introduction of KST may necessitate their immunization (McMahon et al. 2014; Greener et al. 2020). Implementing such strategies, including immunization, is expensive and requires a reliable funding commitment. Ensuring the survival of the KST in its natural habitat will thus strongly depend upon sustained collaborative efforts between the Tanzanian government and other stakeholders, such as NGOs and global conservation partners. Securing the future of the KST population amidst ongoing environmental challenges will thus require a sustained and multifaceted strategy, combining scientific intervention with strong support networks.

In conclusion, our findings highlight the unique nature of the Kihansi Gorge spray wetlands within the Udzungwa Mountains, particularly in how species in this area respond differentially to *Bd*. Some amphibian species that tested positive for *Bd* in Kihansi Gorge spray wetlands were negative in other localities. Therefore, the findings deepen our understanding of the nuanced interactions between *Bd* infection and the toad species and their natural habitats, reaffirm the persistence of *Bd* in the infected species, identify a new *Bd* carrier species, and highlight how *Bd* continues to threaten KST's restoration efforts, even two decades after its initial discovery. Our results, moreover, underscore how habitat contamination with *Bd* can lead to repeated extinctions of captive‐bred KST populations released into the wild, perpetuating high restoration costs over decades. Continuing monitoring and experimentation are therefore essential to adaptively guide conservation efforts to restore this endemic species to its natural habitat in the Kihansi Gorge and reduce the costs associated with maintaining its surging population in captivity.

## Author Contributions

**Devolent T. Mtui:** conceptualization (equal), data curation (lead), formal analysis (lead), investigation (lead), methodology (lead), project administration (lead), resources (lead), supervision (lead), validation (lead), visualization (lead), writing – original draft (lead), writing – review and editing (lead). **Leonard J. Haule:** data curation (equal), investigation (equal), writing – original draft (equal), writing – review and editing (equal). **Joseph O. Ogutu:** formal analysis (lead), writing – original draft (lead), writing – review and editing (lead). **Asa Preston:** formal analysis (equal), resources (equal), writing – review and editing (equal). **Josephine Braun:** formal analysis (equal), resources (equal), writing – review and editing (equal). **William D. Newmark:** methodology (equal). **Edward M. Kohi:** conceptualization (equal). **Juma Kimera:** investigation (equal), methodology (equal). **Mikidadi Mtalika:** investigation (equal). **Hussein Adam:** investigation (equal). **Samueli Mtoka:** investigation (equal). **Felix Shayo:** investigation (equal). **Julius D. Keyyu:** supervision (supporting). **Mariam R. Makange:** formal analysis (equal). **Jean N. Hakizimana:** writing – review and editing (equal). **Gerald Misinzo:** formal analysis (equal), resources (equal). **Eblate E. Mjingo:** funding acquisition (equal).

## Funding

Financial support was provided by the Government of Tanzania through the Ministry of Natural Resources and Tourism.

## Disclosure

Research Permit: The research was conducted under permit #2020435NA2018259 issued by the Tanzania Commission for Science, Technology, and Innovation (COSTECH).

## Conflicts of Interest

The authors declare no conflicts of interest.

## Supporting information


**Figure S1:** Experimental enclosures.
**Figure S2:** Capturing local insects from the spray wetland using emergent traps (the black umbrella like with a bottle cap at the top).
**Table S1:** Detailed information on the 44 samples collected and analyzed in this study, including sample number, species name, type of sample, habitat type where the specimen was collected, age composition and survey time.
**Table S2:** Positive Quantitative Polymerase Chain Reaction Product observed on the basis of Cycle threshold (Ct) values of < 50 on 4 species of amphibians collected from Kihansi Gorge spray wetlands.
**Table S3:** Results of logistic regression relating the occurrence probability of *Batrachochytrium dendrobatidis* to wetland, amphibian species and their interaction. NDF is the numerator and DDF is the denominator degrees of freedom of the F test.

## Data Availability

Field data are embedded in this manuscript as Supporting Information. DNA sequences were submitted to the National Center for Biotechnology Information (NCBI) GenBank and assigned accession numbers PP188411‐13.
