# Supplementary material for: Monitoring the Prevalence and Distribution of Chytrid Fungus (Batrachochytrium dendrobatidis) in the Kihansi Spray Toad (Nectophrynoides asperginis) Population in the Kihansi Gorge Spray Wetlands, Tanzania
Source: Ecol Evol. 2026 Jan 9;16(1):e72873. doi: 10.1002/ece3.72873 (PMC12789641; doi:10.1002/ece3.72873)
Supplement: Supplementary file 1 — Figure S1: Experimental enclosures. Figure S2: Capturing local insects from the spray wetland using emergent traps (the black umbrella like with a bottle cap at the top). Table S1: Detailed information on the 44 samples collected and analyzed in this study, including sample number, species name, type of sample, habitat type where the specimen was collected, age composition and survey time. Table S2: Positive Quantitative Polymerase Chain Reaction Product observed on the basis of Cycle threshold (Ct) values of < 50 on 4 species of amphibians collected from Kihansi Gorge spray wetlands. Table S3: Results of logistic regression relating the occurrence probability of Batrachochytrium dendrobatidis to wetland, amphibian species and their interaction. NDF is the numerator and DDF is the denominator degrees of freedom of the F test. [file ECE3-16-e72873-s001.docx]

**12.0 SUPPLEMENTARY MATERIALS**

Table S1: Detailed information on the 44 samples collected and analyzed in this study, including sample number, species name, type of sample, habitat type where the specimen was collected, age composition and survey time. Note that USW, LSW, MSW and TW represent the upper, lower, and mid spray wetlands, respectively, and TW is a code for TAWIRI, i.e., Tanzania Wildlife Research Institute.

| **Sample No.** | **Species name** | **Type of sample** | **Habitat type** | **Age group** | **Survey time** |
| --- | --- | --- | --- | --- | --- |
| LSW-TWS001 | *Arthroleptides yakusini* | Swabs | On a big rock by the river | Tadpole | Night |
| LSW-TWS002 | *Arthroleptides yakusini* | Swabs | On a big rock by the river | Tadpole | Night |
| LSW-TWS003 | *Arthroleptides yakusini* | Swabs | On a big rock by the river | Tadpole | Night |
| LSW-TWS004 | *Arthroleptides yakusini* | Swabs | On a big rock by the river | Juvenile | Night |
| LSW-TWS005 | *Arthroleptides yakusini* | Swabs | On a big rock by the river | Tad pole | Night |
| LSW-TWS006 | *Arthroleptides yakusini* | Swabs | On vegetation inside the wetland | Adult | Night |
| LSW-TWS007 | *Arthroleptides yakusini* | Swabs | On vegetation inside the wetland | Adult | Night |
| LSW-TWS008 | *Arthroleptides yakusini* | Swabs | On vegetation inside the wetland | Adult | Night |
| LSW-TWS009 | *Arthroleptides yakusini* | Swabs | On vegetation inside the wetland | Adult | Night |
| LSW-TWS0010 | *Arthroleptis xenodactyloides* | Swabs | On top of vegetation inside the wetland | Adult | Night |
| LSW-TWS0011^±^ | *Arthroleptides yakusini* | Swabs | On vegetation inside the wetland | Adult | Night |
| LSW-TWS0012^±^ | *Pytchadena anchietae* | Swabs | By the entrance on grasses | Adult | Night |
| LSW-TWS0013^±^ ⃰⃰ | *Pytchadena anchietae* | Swabs | On the entrance to the wetland at the sterilization point on grasses | Adult | Night |
| LSW-TWS0014 ⃰⃰ | *Pytchadena anchietae* | Swabs | On the entrance to the wetland at the sterilization point on grasses | Adult | Night |
| LSW-TWS0015 | *Afrixalus fornasinii* | Swabs | On vegetation at the entrance of the wetland (sterilization point) | Adult | Day |
| LSW-TWS0017 | *Arthroleptides yakusini* | Swabs | On vegetation inside the wetland | Tad pole | Day |
| USW-TSW0018 | *Hyperolius substriatus* | Swabs | On a false ginger plant close to the edge of the wetland | Adult | Night |
| USW-TSW0019 | *Hyperolius substriatus* | Swabs | On a false ginger plant close to the edge of the wetland | Adult | Night |
| USW-TSW0020 **⃰⃰** | *Hyperolius substriatus* | Swabs | On a false ginger plant close to the edge of the wetland | Adult | Night |
| USW-TSW0021 | *Hyperolius substriatus* | Swabs | On a false ginger plant close to the edge of the wetland | Adult | Night |
| USW-TSW0022 | *Hyperolius substriatus* | Swabs | On a false ginger plant close to the edge of the wetland | Adult | Night |
| USW-TSW0023 | *Hyperolius substriatus* | Swabs | On a false ginger plant close to the edge of the wetland | Adult | Night |
| USW-TSW0024 | *Pytchadena anchietae* | Swabs | Inside the wetland on vegetation | Adult | Night |
| USW-TSW0025 | *Hyperolius substriatus* | Swabs | On a false ginger plant close to the edge of the wetland | Adult | Night |
| USW-TSW0026 | *Hyperolius mitchelli* | Swabs | On a false ginger plant close to the edge of the wetland | Adult | Night |
| USW-TSW0027 | *Arthroleptis xenodactyloides* | Swabs | On top of a very short broadleaf plant under lots of sprinkler water | Adult | Night |
| USW-TSW0028^±^ | *Arthroleptides yakusini* | Swabs | On vegetation inside the wetland | Adult | Night |
| USW-TSW0029 | *Arthroleptides yakusini* | Swabs | On the sprinkler pole inside the wetland | Adult | Night |
| USW-TSW0030 | *Arthroleptides yakusini* | Swabs | On vegetation inside the wetland | Adult | Night |
| USW-TSW0031 **⃰⃰** | *Arthroleptides yakusini* | Swabs | On a big rock (dry part of rock) | Adult | Night |
| USW-TSW0032^±^ **⃰⃰** | *Arthroleptides yakusini* | Swabs | On a big rock (dry part of rock) | Adult | Night |
| USW-TSW0033 **⃰⃰** | *Pytchadena anchietae* | Swabs | On the entrance to the wetland at the sterilization point on grasses | Adult | Night |
| USW-TSW0035 **⃰⃰** | *Arthroleptides yakusini* | Swabs | On a rock inside the wetland (lots of water splash) | Adult | Night |
| MSW-TWS0036 | *Nectophrynoides asperginis* | Swabs | On top of broadleaf plant splashed with lots of sprinkler water inside the wetland | Adult | Night |
| MSW-TWS0037 **⃰⃰** | *Nectophrynoides asperginis* | Swabs | Inside the wetland (dead in experimental cage- one of the F1 generation) | Adult | Night |
| MSW-TWS0038 | *Arthroleptides yakusini* | Swabs | On rock by the edge of wetland but reached with water | Adult | Night |
| MSW-TWS0039 | *Arthroleptides yakusini* | Swabs | On rock by the edge of wetland but reached with water | Adult | Night |
| MSW-TWS0040 **⃰⃰** | *Arthroleptides yakusini* | Swabs | Inside the wetland under vegetation with lots of sprinkler water | Adult | Night |
| MSW-TWS0041 | *Arthroleptides yakusini* | Swabs | Inside the wetland under lots of sprinkler water | Adult | Night |
| MSW-TWS0042 **⃰⃰** | *Arthroleptides yakusini* | Swabs | On a rock inside the wetland (lots of water splash) | Adult | Night |
| MSW-TWS0043 | *Afrixalus fornasinii* | Swabs | On vegetation by the edge of the wetland first pole of sprinklers) | Adult | Night |
| MSW-TWS0045 **⃰⃰** | *Nectophrynoides asperginis* | Complete specimens | Five dead toads inside experimental cage No. 4 | Sub-adults (8 months) | Preserved in 70% Ethanol |
| MSW-TWS0046 **⃰⃰** | *Nectophrynoides asperginis* | Complete specimens | Five dead toads inside experimental cage No. 8 | Adult | Preserved in 70% Ethanol |
| MSW-TWS0047 **⃰⃰** | *Nectophrynoides asperginis* | Complete specimens | Five dead toads inside experimental cage No. 10 | Adult | Preserved in 70% Ethanol |

[Note: Samples with symbols ± and ⁎ symbols tested positive to *Bd* using conventional and quantitative Polymerase Chain Reaction respectively]

Table S2. Positive Quantitative Polymerase Chain Reaction Product observed based on Cycle threshold (Ct) values of < 50 on 4 species of amphibians collected from Kihansi Gorge spray wetlands. [Note: rep. = replication; conf. = confirmation; Av. = average]

| **Animal Id** | **Species** | **Ct rep.1** | **Ct rep.2** | **Ct rep.3** | **Av. ITS copies per well** | **Ct rep.1 conf.** | **Ct rep.2 conf.** | **Ct rep.3 conf.** | **Ct rep.4 conf.** | **Ct rep.5 conf.** | **Av. ITS copies per well** |
| --- | --- | --- | --- | --- | --- | --- | --- | --- | --- | --- | --- |
| TWS0013 | *Pytchadena anchietae* | 28.99 | 28.98 | 28.91 | 3204.86 | . | . | . | . | . | . |
| TWS0014 | *Pytchadena anchietae* | 0.00 | 39.22 | 41.10 | 1.93 | . | . | . | . | . | . |
| TWS0033 | *Pytchadena anchietae* | 0.00 | 0.00 | 40.26 | 1.49 | 39.32 | 38.51 | 0.00 | 38.34 | 0.00 | 4.66 |
| TWS0031 | *Arthroleptides yakusini* | 37.95 | 40.25 | 37.68 | 5.74 | . | . | . | . | . | . |
| TWS0032 | *Arthroleptides yakusini* | 36.61 | 37.01 | 36.50 | 16.79 | . | . | . | . | . | . |
| TWS0035 | *Arthroleptides yakusini* | 33.12 | 33.51 | 33.22 | 170.85 | . | . | . | . | . | . |
| TWS0040 | *Arthroleptides yakusini* | 38.97 | 40.21 | 38.70 | 3.14 | . | . | . | . | . | . |
| TWS0042 | *Arthroleptides yakusini* | 39.65 | 41.20 | 0.00 | 1.52 | . | . | . | . | . | . |
| TWS0020 | *Hyperolius substriatus* | 42.19 | 53.99 | 0.00 | 0.20 | 40.51 | 40.13 | 39.05 | 0.00 | 0.00 | 1.83 |
| TWS0037 | *Nectophrynoides asperginis* | 24.54 | 24.60 | 24.52 | 63856.07 | . | . | . | . | . | . |
| TWS0045 | *Nectophrynoides asperginis* | 21.94 | 21.87 | 21.90 | 386337.00 | . | . | . | . | . | . |
| TWS0046 | *Nectophrynoides asperginis* | 23.32 | 23.23 | 23.43 | 147052.86 | . | . | . | . | . | . |
| TWS0047 | *Nectophrynoides asperginis* | 26.70 | 26.57 | 26.97 | 13688.09 | . | . | . | . | . | . |

Table S3. Results of logistic regression relating the occurrence probability of *Batrachochytrium dendrobatidis* to wetland, amphibian species and their interaction. NDF is the numerator and DDF is the denominator degrees of freedom of the F test.

| **Effect** | **NDF** | **DDF** | **F-Value** | **Pr > F** |
| --- | --- | --- | --- | --- |
| Wetland | 2 | 76 | 0 | 1 |
| Amphibian species | 6 | 76 | 0 | 1 |
| Wetland*Amphibian species | 3 | 76 | 0 | 1 |

**
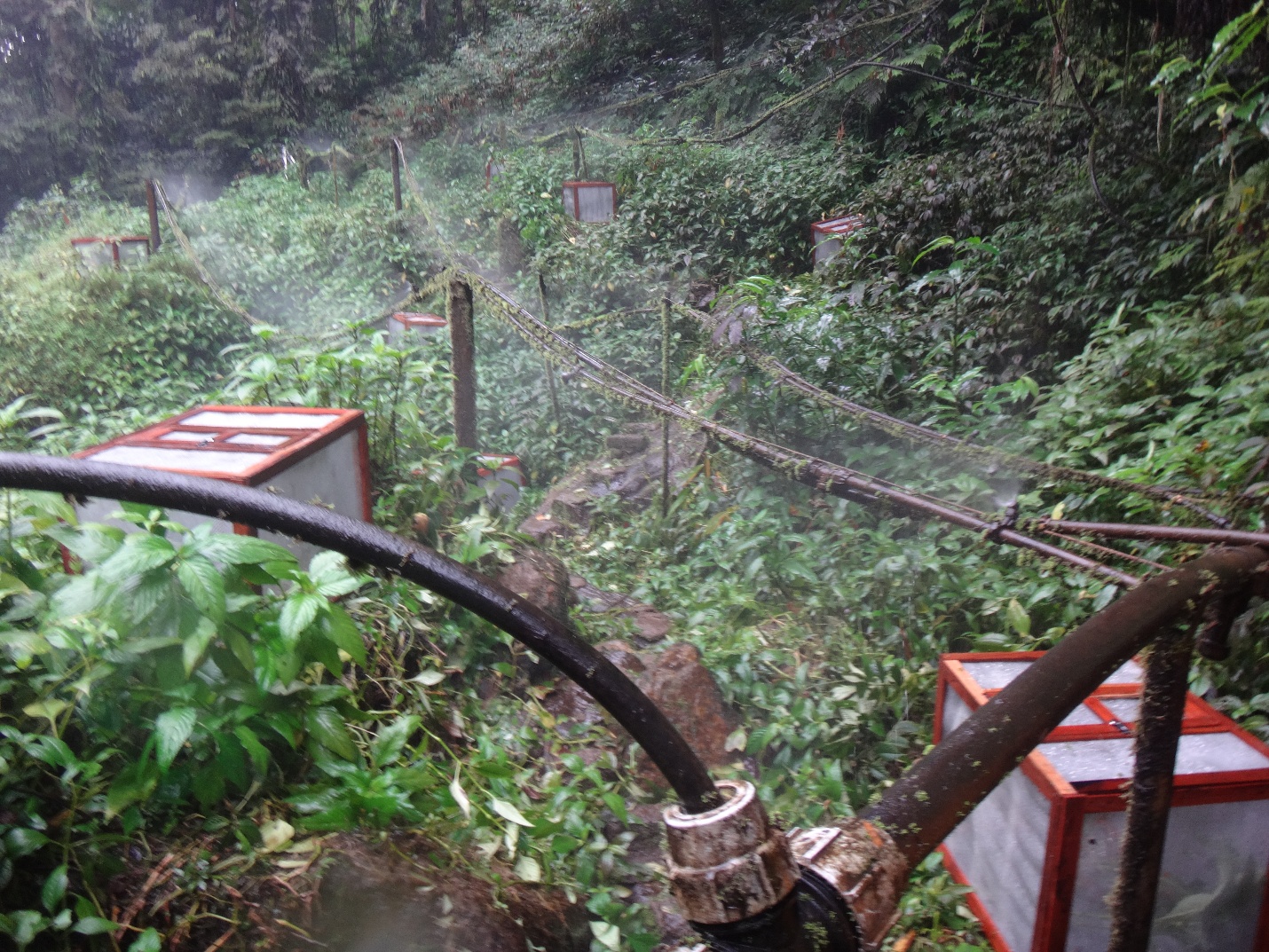
**

Supplementary Figure 1

Figure S 1. Experimental enclosures

**
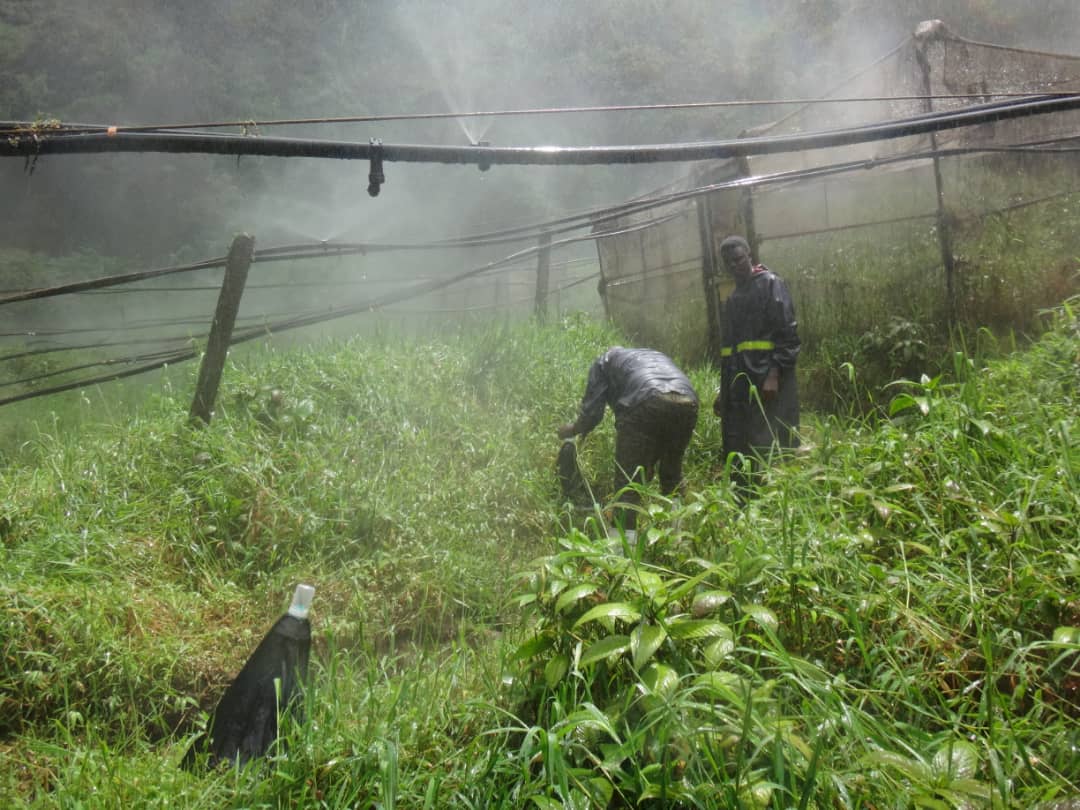
**

Figure S 2. Capturing local insects from the spray wetland using emergent traps (the black umbrella like with a bottle cap at the top)
